# Comparison of Postoperative Analgesic Effect of Intrathecal Clonidine and Fentanyl Added to Bupivacaine in Patients Undergoing Cesarean Section: A Prospective Randomized Double-Blind Study

**DOI:** 10.1155/2014/513628

**Published:** 2014-02-04

**Authors:** Marzieh Beigom Khezri, Meisam Rezaei, Morteza Delkhosh Reihany, Ezzatalsadat Haji Seid Javadi

**Affiliations:** ^1^Department of Anesthesiology, Faculty of Medicine, Qazvin University of Medical Sciences, Shahid Bahonar Boulevard, P.O. Box 34197/59811, Qazvin 3419759811, Iran; ^2^Department of Obstetrics and Gynecology, Faculty of Medicine, Qazvin University of Medical Sciences, Shahid Bahonar Boulevard, P.O. Box 34197/59811, Qazvin 3419759811, Iran

## Abstract

*Objectives.* To compare the analgesic efficacy of intrathecal clonidine and fentanyl added to bupivacaine after cesarean section. *Methods.* Ninety patients scheduled for cesarean section under spinal anesthesia were randomly allocated to one of the three following groups to receive bupivacaine 10 mg combined with 75 *µ*g clonidine (group C), bupivacaine 10 mg combined with 0.5 mL fentanyl (group F), and bupivacaine 10 mg combined with 0.5 mL distilled water (group P), intrathecally. The time to first analgesic request, analgesic requirement in the first 24 hours after surgery, sensory and motor blockade onset time, duration of sensory and motor blockade, the incidence of hypotension, ephedrine requirements, bradycardia, and hypoxemia were recorded. *Results.* The duration of anesthesia in clonidine group (275.10 ± 96.09) was longer compared to the placebo (211.73 ± 74.80) and fentanyl (192.33 ± 30.36) groups. This difference between group C versus F (*P* = 0.006) and P groups (*P* < 0.001) was significant. Similarly, the mean time to first analgesic request was also longer in group C (519.44 ± 86.25) than in groups F (277.88 ± 94.25) and P (235.43 ± 22.35 min). This difference between group C versus F (*P* < 0.001) and P groups (*P* < 0.001) was significant. *Conclusion.* Intrathecal clonidine 75 *µ*g with bupivacaine prolonged the time to first analgesic request compared to fentanyl; however, the total analgesic consumption within the first 24 h postoperative was similar in fentanyl and clonidine groups following cesarean section. This trial is registered with ACTRN12611000909921 and ClinicalTrials.gov NCT01425658.

## 1. Introduction

Pain control after cesarean improves breastfeeding and satisfaction of mother. In addition, inadequate analgesia leads to elevated plasma catecholamine concentrations, resulting in adverse effect on all organ systems [1]. Neuraxial analgesia using only local anesthetic often provides suboptimal analgesia with higher side effects. Many drugs have been adjusted to local anesthetics to provide optimal analgesia with lower side effects such as opioids, epinephrine, ketamine, midazolam, clonidine, and magnesium [2, 3]. Opioids are usually used for providing better analgesia and reducing the side effects. Fentanyl exhibits close structural similarities to local anesthetics and has demonstrable local anesthetic effect on sensory C primary afferent nerve fibers, which may facilitate analgesic effects [4, 5]. Furthermore, fentanyl is the most frequently intrathecal lipophilic opioid used as analgesic agent with minimal cephalad spread making it the least likely of all the intrathecal opioids to cause delayed respiratory depression [5]. However, in parturients, the advantageous analgesia has to be balanced against maternal and fetal side effects such as bradycardia, respiratory depression, arterial hypotension, nausea, vomiting, and pruritus. Furthermore, it is reported that a single administration of an opioid may also induce a long lasting increase of threshold pain sensitivity, leading to delayed hyperalgesia [6]. On the contrary, it is reported that clonidine by stimulation of *α*_2_ adrenoreceptors beyond the analgesic effects possesses antihyperalgesic properties [7–9]. Clonidine mimics the effects of norepinephrine and it antihyperalgesic mechanisms that partly depend on fortification of noradrenergic inhibitory controls in the dorsal horn of the spinal cord [10]. The safety of intrathecal clonidine has been extensively evaluated in animals, humans, and obstetrical anesthesia [6, 11–16]. Also it is reported that clonidine administrated via intrathecal route was undetectable in the fetal circulation with no obvious effect on the neonatal Apgar scores [12, 16].

We hypothesized that clonidine may provide a better pain relief after cesarean section compared to fentanyl. In addition, unlike spinal opioids, clonidine does not produce pruritus, hyperalgesia, or respiratory depression. To test our hypothesis, we designed this randomized-double-blind, placebo-controlled study to compare the postoperative analgesic effect of intrathecal clonidine and fentanyl added to bupivacaine in patients undergoing cesarean section.

## 2. Methods

**Figure 1:**
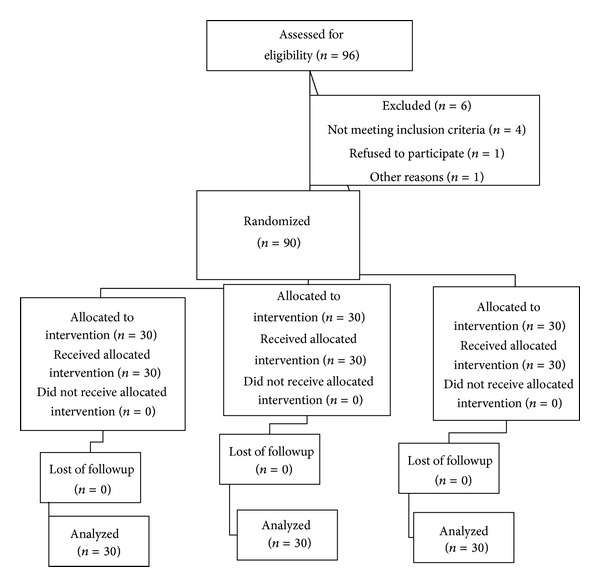
Consort flow of diagram.

After approval of the Institutional Ethical Committee and written informed consent, Ninety-six patients 18–45 years old ASA physical status I or II, scheduled for cesarean section under spinal anesthesia, were enrolled in a prospective, double-blind, randomized parallel study. The recommendations by the Consolidated Standards of Reporting Trials (CONSORT) for reporting a randomized, controlled clinical trial [17] were followed (Figure 1). Exclusion criteria included significant coexistence of conditions such as hepatorenal and cardiovascular diseases, any contraindication to regional anesthesia such as local infection or bleeding disorders, allergy to bupivacaine or clonidine, long-term opioid use, or a history of chronic pain. The patients were randomly allocated to one of three groups of 30 members each by using the computer-generated randomization list. Blinding was achieved through the use of equal amounts of drugs (2.5 mL), while each syringe was labeled as A, B, and C according to its contents. Identical coded syringes prepared by the personnel not involved in the study were randomly handed to the anesthetists, who were unaware of the identity of the drugs. The clonidine group received bupivacaine 10 mg combined with 75 *μ*g of preservative free clonidine; the fentanyl group received bupivacaine 10 mg combined with 25 *μ*g fentanyl; and the placebo group received bupivacaine 10 mg combined with 0.5 mL distilled water, intrathecally. All patients received an intravenous preload of 5–7 mL/kg lactated Ringer's solution before a subarachnoid block. Later, using an aseptic technique, a 25-gauge Quincke needle was inserted intrathecally via a midline approach into the L4-5 interspaces by the anesthetist who was unaware of patient assignment while the patient was in sitting position. After a successful dural puncture, the anesthetic solution was injected. The primary outcomes of this randomized, double-blind and placebo-controlled clinical trial are to evaluate the time to first requirement of analgesic supplement and total analgesic consumption in the first 24 h postoperative. The secondary outcomes included the assessment of sensory block onset time, onset of motor block, duration of blockade, hemodynamic variables, the incidence of hypotension, ephedrine requirements, bradycardia, hypoxemia (saturation of peripheral oxygen (SpO_2_) < 90), and adverse events such as sedation, dizziness, pruritus, and postoperative nausea and vomiting.

In this study, the postoperative analgesia was defined as the time to first requirement of analgesic supplement from the time of injection. No additional analgesic was administered unless requested by the patient. Sensory block was assessed by a pinprick test. The onset of sensory block was defined as the time between the end of injection of the intrathecal anesthetic and the absence of pain at the T10 dermatome; the duration of sensory block was defined as the time for regression of the sensory from the maximum block height to the T10 dermatome as evaluated by pinprick. The maximum level of sensory block was evaluated by pinprick after 20 min following completion of injection. Motor block was assessed by the modified Bromage score (0: no motor loss; 1: inability to flex the hip; 2: inability to flex the knee; and 3: inability to flex the ankle); the onset of motor block was defined as the time from intrathecal injection to Bromage block 1, whereas the duration of motor block was assumed when the modified Bromage score was zero. The duration of spinal anesthesia was defined as the period from spinal injection to the first occasion when the patient complained of pain in the postoperative period. Patients were preoperatively instructed to use the verbal rating scale (VRS) from 0 to 10 (0: no pain, and 10: maximum imaginable pain) for pain assessment. If the VRS exceeded four and the patient requested a supplement analgesic, diclofenac Na sup. 100 mg every 8 hours was given to relieve the postoperative pain as needed (q 8 h PRN). If the time course following the administration of diclofenac Na decreased to less than 8 h and the patient made another request for supplement analgesic, pethidine 25 mg IV was given.

The mean arterial pressure (MAP), heart rate (HR), and peripheral oxygen saturation (SpO_2_) were recorded by an anesthetist blinded to the patient group 5 min before the intrathecal injection and also 2, 4, 6, 8, 10, 15, and 20 min after injection. If systolic blood pressure (SBP) was 20% below the baseline (5 min before the intrathecal injection) or less than 90 mmHg, ephedrine 5 mg was administered intravenously. Also, if HR was less than 50 beats/min, 0.5 mg of atropine sulfate was administered intravenously. A follow-up telephone call was made 24 h after surgery and again 1 and 6 months later, during which the patients were asked about the side effects and dysesthesia of the lower limbs or buttocks. To calculate the sample size, data from previous similar studies were taken into consideration [2, 3, 12, 14]. A sample size of 25 patients per group was required to detect a 20 min difference in the median duration of analgesia between the groups using the Mann-Whitney *U* test, with a power of 0.9 and an *α* equal to 0.05. We included 30 patients in each group to allow for dropouts and protocol violations. Data were analyzed using SPSS (SPSS 15.0, SPSS Inc, Chicago, IL, USA). Continuous variables were tested for normal distribution by the Kolmogorov-Smirnov test. Parametric data were expressed as mean and standard deviation (SD) and analyzed by independent *t*-test. Nonparametric data were expressed as median and interquartile range (IQR) and analyzed using the Mann-Whitney *U* test. The effect of time on hemodynamic parameters was analyzed using repeated measurement analysis of variance. The *χ* ^2^ test was used to analyze the incidence of side effects. Pain scores, motor scores, and sensory level were evaluated within the groups using the Wilcoxon's signed rank test. A *P* value <0.05 was considered as significant, statistically.

## 3. Results

A total of 96 patients initially enrolled in this study, 6 patients had to be excluded because of logistical reasons or other violations of the study protocol. Ninety patients were included and randomly assigned to their treatment groups (Figure 1).

There were no significant differences in age, height, and weight among the three groups. The duration of surgery was also similar (Table 1).

The mean onset of sensory block was 90 ± 23 sec in group C 95.33 ± 39.17 sec in group F and 78.5 ± 26.00 sec in group P. The difference between group C versus group F (*P* = 0.523) and P (0.075) was insignificant. Similarly, this difference in groups F and P was also insignificant (*P* = 0.055). The mean duration of sensory block in group C (169.66 ± 25.69 min) was longer than group F (122.23 ± 32.78 min) and group P (133.53 ± 32.68 min). The difference between group C versus group F (*P* < 0.001) and P (*P* < 0.001) was significant, but the difference between groups F and P (*P* = 0.186) was found to be insignificant. The mean onset of motor block was 81.33 ± 26.71 in group C, 80.00 ± 30.62 in group F, and 81.83 ± 27.21 sec in group P. The difference between group C versus group F (*P* = 0.858) and P (*P* = 0.943) was insignificant. Similarly, the difference in groups F and P was insignificant (*P* = 0.807). The median value found for the maximum height of block was T6 for all three groups. The mean duration of motor blockade time was significantly longer in group C (182.66 ± 33.12 min) than F (136.76 ± 28.85 min) and P groups (143.16 ± 33.94). The difference in mean duration of motor blockade time between group C versus F (*P* < 0.001) and P groups (*P* < 0.001) was significant whereas no significant difference in duration of motor block between F and P groups was found (*P* = 0.435). The duration of anesthesia in clonidine group (275.10 ± 96.09) was longer compared to the placebo (211.73 ± 74.80) and fentanyl (192.33 ± 30.36) groups. As shown in Table 2, the patients who were given clonidine had a significantly prolonged duration of anesthesia compared with control (*P* < 0.001) and F groups (*P* = 0.006). As to the duration of anesthesia, the mean time to first analgesic request was also significantly longer in group C (519.44 ± 86.25) than in groups F (277.88 ± 94.25) and P (235.43 ± 22.35 min). This difference between group C versus F (*P* < 0.001) and P groups (*P* < 0.001) was significant. Likewise, the difference between groups F and P was also significant (*P* = 0.022). The total number of analgesic request by patients during 24 hours after surgery in clonidine group was significantly smaller than in control group (*P* = 0.002). Total analgesic consumption during 24 hours after surgery failed to demonstrate a significant difference between F and C groups (*P* = 0.318).

**Figure 2:**
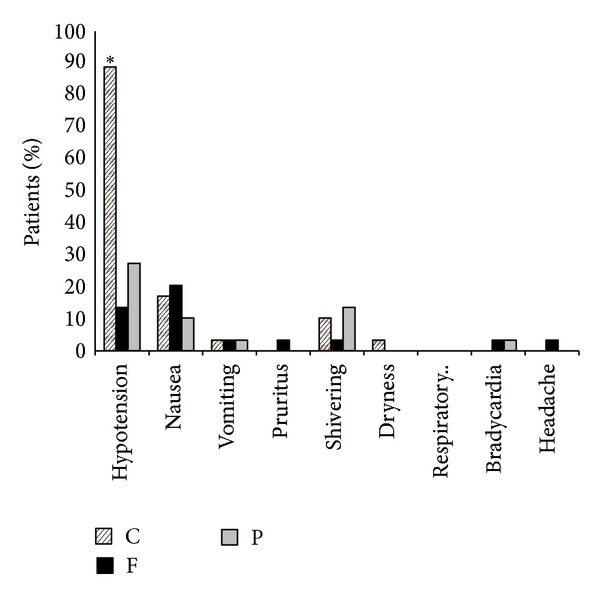
Side effects observed in three study groups. C: clonidine, F: fentanyl, and P: placebo. *Significant difference between the three groups.

As shown in Table 3, the mean variation of mean arterial pressure and heart rate was defined as the difference between the highest and the lowest mean arterial pressure and heart rate in each patient. The mean variation of MAP was 50.70 ± 21.65 in group C, 33.73 ± 10.73 in group P, and 50.00 ± 76.14 in group F. This difference between group C versus P (*P* < 0.001) was significant whereas no significant difference between F versus C (*P* = 0.962) and P (*P* = 0.251) groups was found. The overall difference in ephedrine requirement between the three groups was significant, statistically (*P* < 0.001). The mean variation of HR was 34.33 ± 9.7 in group C, 33.43 ± 10.73 in group P, and 32.86 ± 10.17 in group F. The difference between group C versus P (*P* = 0.761) and F (*P* = 0.571) groups was also insignificant as it was for the difference between groups F and P (*P* = 0.851). As shown in Figure 2, the three groups were found to have no significant difference in terms of other intraoperative and postoperative side effects including pruritus, nausea, vomiting, headache, shivering, and respiratory depression. No patient in either group showed any sensory or motor complications within the next six months followup after surgery. All newborns in our study were free of any adverse effect.

## 4. Discussion

Based on the data found in our study, it was concluded that administration of intrathecal clonidine 75 *μ*g with bupivacaine prolonged intraoperative anesthesia and the time to first analgesic request after cesarean delivery compared to fentanyl and control groups. These findings are consistent with previous studies [18, 19]. Analgesic properties of clonidine have been shown to depend on the activation of *α*_2_ receptors located in the dorsal horn. Presynaptic stimulation of *α*_2_ receptors inhibits neurotransmitter release and postsynaptic stimulation prevents neuronal transmission through hyperpolarisation [10].

The second observation which should be emphasized is that although intrathecal clonidine 75 *μ*g with bupivacaine prolonged intraoperative anesthesia and the time to first analgesic request compared to fentanyl yet the total analgesic consumption in the first 24 h postoperative was similar in fentanyl and clonidine groups after elective cesarean delivery. The possible explanation for this finding is that the analgesic effect of clonidine follows a dose-dependent manner. Eisenach et al. reported that a dose of 150 *μ*g clonidine is required to observe antihyperalgesic effect, while a lower dose (50 *μ*g) is ineffective [8, 18, 20]. The selected dose of intrathecal clonidine in current study was based on several reasons. Firstly, intrathecal clonidine displays the risk of adverse intraoperative hemodynamic effects. Rochette et al. showed that clonidine at a dose of 1 *μ*g/kg was not associated with hemodynamic disturbance [21]. Also, Bajwa et al. [22] found that the optimal dose for clonidine to produce effective analgesia without inducing hypotension in emergency cesarean section is 37.5 *μ*g. However, most studies have reported that although clonidine at a lower intrathecal dose less than 0.5 *μ*g/kg body weight was devoid of its diverse side effects, at the same time the antinociceptive effect of this drug was also reduced significantly [7, 12–14, 16]. Secondly, it is reported that intrathecal clonidine possesses an analgesic plateau effect at 75 *μ*g and higher doses could only increase the duration but not the intensity of analgesia [8].

The third finding which should be considered is that intrathecal clonidine clearly increases the duration of both sensory block and motor block as well as postoperative pain relief. This finding is also consistent with the previous studies [18, 23, 24]. The mechanism of clonidine-induced potentiation of sensory block in spinal anesthesia is reported to be dependent on presynaptic (decrease in transmitter release) and postsynaptic (increase in hyperpolarization) action [25, 26].

The fourth finding which should be taken into account is that transient hypotension episodes and vasopressor requirement in clonidine group were significantly greater than F and P groups, a finding in agreement with previous studies [27, 28]. Except for sympatholytic action of clonidine and profound analgesia which also reduces sympathetic activity, no other clear explanation is available. In contrast, some studies have reported that clonidine at doses between 37.5 and 150 *μ*g failed to cause a significant decrease in blood pressure when added to a high dose of bupivacaine (18 mg) [13, 29]. However, these apparently controversial findings may be due to either the difference in bupivacaine and clonidine doses or dissimilarity in population and the type of surgeries. The fifth observation which should be noted is that clonidine lacks the ability to prevent postspinal shivering; by contrast, it is confirmed that clonidine, when administered intravenously, is an effective drug to prevent shivering in patients undergoing spinal anesthesia [30, 31], a finding compatible with that found in a study by Jeon et al. [32]. The possible reason for this finding could be attributed to the inability of clonidine to inhibit afferent thermal conduction at the level of spinal cord. All newborns in our study were free of any adverse effect. We concluded that intrathecal clonidine 75 *μ*g with bupivacaine prolonged intraoperative anesthesia and the time to first analgesic request compared to fentanyl, however, the total analgesic consumption in the first 24 h postoperative was similar in fentanyl and clonidine groups following elective cesarean delivery. Further studies are needed to evaluate the analgesic efficacy of clonidine with other neuraxial drug combinations such as epinephrine, ketamine, and magnesium to provide better analgesia and reduce the incidence and severity of side effects.

## Figures and Tables

**Table 1:** Demographic data for three study groups.

Groups	Group C (*n* = 30)	Group F (*n* = 30)	Group P (*n* = 30)
Age (years)	30.43 ± 3.70	30.20 ± 5.41	29.16 ± 5.11
Weight (kg)	88.5 ± 15.4	88.5 ± 13.6	89.7 ± 11.9
Height (cm)	166 ± 4.6	160 ± 8.4	162 ± 6.1
Duration of surgery (min)	85.63 ± 15.70	79.16 ± 20.11	81.70 ± 18.76

Values are presented as mean ± SD. C: clonidine, F: fentanyl, and P: placebo. There are no significant differences among the three groups.

**Table 2:** Characteristics of spinal anesthesia.

Groups	Group C (*n* = 30)	Group F (*n* = 30)	Group P (*n* = 30)	*P*
Onset time of sensory block (second)	90 ± 23	95.33 ± 39.17	78.5 ± 26.00	NS
Duration of sensory block (min)	169.66 ± 25.69	122.23 ± 32.78	133.53 ± 32.68	<0.001
Onset time of motor block (second)	81.33 ± 26.71	80.00 ± 30.62	81.83 ± 27.21	NS
Duration of motor block (min)	182.66 ± 33.12	136.76 ± 28.85	143.16 ± 33.94	<0.001
Time to first request of analgesic (min)	519.44 ± 86.25	277.88 ± 94.25	235.43 ± 22.35	<0.001
Duration of spinal anesthesia	275.10 ± 96.09	211.73 ± 74.80	192.33 ± 30.36	<0.001
Total ephedrine requirement	10.83 ± 5.26	4.16 ± 5.84	2.16 ± 5.52	<0.001
Total analgesic consumption in 24 h (number of analgesic request)	2 (2-2)	2 (1–3)	3 (2-3)	0.011

Values are presented as mean ± SD or median IQR C: clonidine, F: fentanyl, and NS: nonsignificant (*P* > 0.05).

**Table 3:** Hemodynamic variables in the three groups.

Groups	Group C (*n* = 30)	Group P (*n* = 30)	Group F (*n* = 30)	*P*
MAP 5 min before SA	97.23 ± 7.62	94.76 ± 8.82	95.05 ± 5.36	NS
MAP 2 min after SA	73.25 ± 17.46	85.78 ± 11.13	74.76 ± 15.95	0.003
MAP 4 min after SA	69.54 ± 15.39	79.57 ± 11.69	74.93 ± 13.71	0.021
MAP 6 min after SA	89.84 ± 34.82	73.98 ± 13.39	82.12 ± 12.61	0.030
MAP 8 min after SA	91.72 ± 20.19	79.08 ± 13.00	87.54 ± 10.57	0.006
MAP 10 min after SA	92.96 ± 16.70	75.63 ± 12.55	89.11 ± 7.93	<0.001
MAP 15 min after SA	92.52 ± 17.22	78.92 ± 11.95	92.66 ± 9.09	0.001
MAP 20 min after SA	87.20 ± 15.81	78.14 ± 8.94	107.70 ± 76.69	0.041
MAP 25 min after SA	83.64 ± 17.55	75.21 ± 9.31	86.38 ± 4.66	0.001
MAP 30 min after SA	78.43 ± 13.34	66.58 ± 8.87	89.73 ± 5.56	<0.001
HR 5 min before SA	99.40 ± 11.88	97.90 ± 15.40	98.73 ± 13.61	NS
HR 2 min after SA	96.46 ± 16.05	101.20 ± 19.13	101.73 ± 16.15	NS
HR 4 min after SA	89.13 ± 13.62	96.46 ± 17.38	97.26 ± 15.03	NS
HR 6 min after SA	87.50 ± 13.43	97.16 ± 19.61	98.90 ± 17.58	0.024
HR 8 min after SA	84.40 ± 10.87	95.06 ± 20.14	92.73 ± 17.36	0.037
HR 10 min after SA	87.36 ± 13.29	96.23 ± 22.76	91.70 ± 13.68	NS
HR 15 min after SA	83.46 ± 10.48	101.76 ± 17.82	89.13 ± 14.32	<0.001
HR 20 min after SA	85.56 ± 12.38	102.26 ± 20.37	88.26 ± 14.49	<0.001
HR 25 min after SA	88.73 ± 11.46	99.50 ± 18.04	90.86 ± 12.33	0.011
HR 30 min after SA	86.76 ± 9.20	97.83 ± 17.07	89.50 ± 11.36	0.004

Data are presented as mean ± SD. C: clonidine, F: fentanyl, P: placebo, MAP: mean arterial blood pressure (mm Hg), HR: heart rate (bpm), SA: spinal anesthesia, and NS: nonsignificant.
